# Another Fanconi anemia gene joins the club

**DOI:** 10.1172/JCI192382

**Published:** 2025-06-02

**Authors:** Claire C. Homan, Hamish S. Scott, Parvathy Venugopal

**Affiliations:** 1 Centre for Cancer Biology, SA Pathology and University of South Australia, Adelaide, South Australia, Australia.; 2 Adelaide Medical School, The University of Adelaide, Adelaide, South Australia, Australia.; 3 Department of Genetics and Molecular Pathology, SA Pathology, Adelaide, South Australia, Australia.

## Abstract

Fanconi anemia (FA) is the most common inherited bone marrow failure disorder, caused by pathogenic variants in genes involved in the FA DNA repair pathway. In this issue of the *JCI*, two studies report three germline homozygous loss-of-function variants in *FAAP100*, a key component of the FA core complex, identified in three unrelated families. These variants result in severe developmental phenotypes that are among the most extreme reported in FA to date. Harrison et al. described individuals from two families with recurrent pregnancy loss and neonatal death due to homozygous *FAAP100* frameshift and truncating variants, respectively. Kuehl et al. identified a homozygous missense variant in a fetus with congenital malformations consistent with FA. Collectively, both studies provide robust functional evidence from ex vivo and in vitro assays with animal models supporting the pathogenicity of these variants and establish *FAAP100* as a causative FA gene.

## Fanconi anemia DNA repair pathway

**Figure 1:**
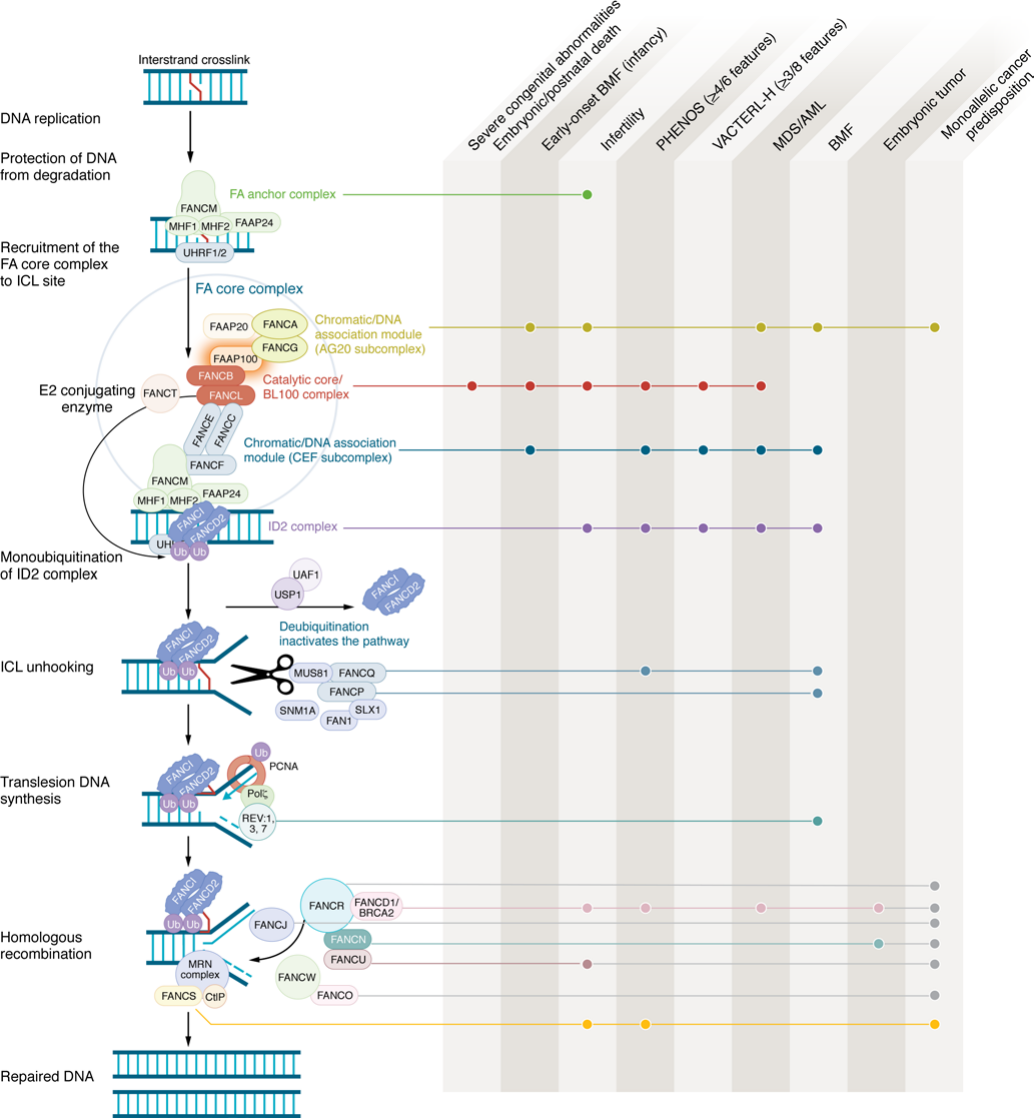
Disruption of the FA/BRCA DNA repair pathway correlates with clinical phenotypes. The FA DNA repair pathway is essential for ICL repair. Upon encountering an ICL, replication forks stall, triggering pathway activation. Subsequently, the anchor complex — comprising FANCM, FAAP24, MHF1, and MHF2 — recognizes the ICL, recruiting the FA core complex to the chromatin. The core complex consists of three distinct subcomplexes: (a) the AG20 complex (FANCA, FANCG, and FAAP20), (b) the BL100/catalytic core complex (FANCB, FANCL, and FAAP100), and (c) the CEF subcomplex (FANCC, FANCE, and FANCF). Among these, the catalytic core complex is essential for E3 ubiquitin ligase activity of the complex. In coordination with FANCT, an E2 ubiquitin–conjugating enzyme, the core complex catalyzes the monoubiquitination of the ID2 complex, a pivotal activation event in the FA repair pathway, which can be regulated by the deubiquitinating enzymes USP1 and UAF1. The monoubiquitinated ID2 complex aids recruitment of structure-specific endonucleases, which mediate ICL unhooking through single-strand DNA cutting. Following unhooking, specialized translesion synthesis (TLS) polymerases perform bypass synthesis across the lesion, leading to the formation of a DNA double-strand break (DSB). This DSB is subsequently repaired through homologous recombination, restoring genomic integrity. Genotype-phenotype correlations of the FA syndrome corresponding to the FA complex genes are supported by strong cohort-based evidence but do not necessarily encompass all reported phenotypes due to clinical heterogeneity within each syndrome. VACTERL-H (vertebral anomalies, anal atresia, cardiac defects, tracheoesophageal fistula, esophageal or duodenal atresia, renal abnormalities, limb defects, and hydrocephalus); PHENOS (Pigmentation, small Head, small Eyes, Neurologic, Otologic, Short stature); MRN, complex including Mre11, Rad50, and Nbs1; Ub, ubiquitin.

Fanconi anemia (FA) is a rare, predominantly autosomal recessive disorder characterized by congenital abnormalities, bone marrow failure (BMF), cancer predisposition, and reproductive defects. The median age of BMF is 7 years, with a reported cumulative incidence of 90% by age 40 (1). Patients exhibit hypersensitivity to DNA crosslinking agents and have an increased risk of hematologic and solid tumors (1, 2). FA results from germline variants in the FA/breast cancer (FA/BRCA) DNA repair pathway, which is essential for interstrand crosslink (ICL) repair. This pathway can be categorized into three functional groups: (a) upstream members***,*** which recognize DNA damage and form the FA core complex, facilitating monoubiquitination of (b) the FANCD2-FANCI heterodimer (ID2) complex, and subsequent activation of (c) downstream factors recruited to repair the DNA damage (Figure 1). Within the 27 known components of this pathway, pathogenic variants in 22 genes have been identified as cause of FA. Harrison et al. (3) and Kuehl et al. (4) identified three families with homozygous FAAP100 variants. Seventeen years ago, the FAAP100 was initially recognized for its role in stabilizing the FA core complex and promoting FANCD2 monoubiquitination. FAAP100 knockdown in cells exhibited hallmark FA characteristics, including hypersensitivity to DNA crosslinking agents and mitomycin C–induced (MMC-induced) chromosomal breakage (5). Now, the first human cases confirm its role in FA pathogenesis, underscoring the critical role of the catalytic core (composed of FANCB, FANCL, and FAAP100) in FA regulation and earning FAAP100 its formal designation as “FANCX,” the newest FANC gene family member (3, 4).

## FA core complex in FA pathogenesis

The FA core complex is assembled at the ICL site, triggering the DNA repair signaling cascade via monoubiquitination of the ID2 complex. The catalytic core is the minimal module required for monoubiquitination, while the other members contribute to maximal activity and specificity of the pathway (6, 7). The number of patients with described mutations for each FA core complex component varies widely. FANCA causes 60%–70% of FA cases and has substantial clinical heterogeneity, with many patients presenting milder phenotypes, having prolonged survival into adulthood, and later-onset myelodysplastic syndrome (MDS) and acute myeloid leukemia (AML) (8, 9). FANCC and FANCG account for 20% of FA cases, while mutations in the remaining 20 FANC genes are extremely rare (i.e., 0.1%–4%) (2). This may be due to their recent discovery and/or because they result in severe, fetus-lethal phenotypes that limit the affected gene within the population, both possibly leading to underdiagnoses.

## Establishing genotype-phenotype correlations

The complexity and redundancies of the FA pathway and the rarity of pathogenic mutations in many FA genes, combined with variable clinical presentations, make genotype-phenotype correlations difficult to establish. Typically, mutations in downstream components cause a less severe phenotype (8, 10), whereas those in the core complex, particularly the catalytic core, lead to more severe congenital presentations (Figure 1). Missense variants within FA complement groups typically cause milder phenotypes than truncating variants, supporting autosomal recessive inheritance with loss-of-function mutations, with missense alleles being hypomorphs (8, 10, 11). The studies by Harrison et al. (3) and Kuehl et al. (4), present three families homozygous for different FAAP100 variants. To date, the severity of FAAP100 FA has been found to be similar to that caused by its fellow catalytic core components, FANCB and FANCL, resulting in multiple severe congenital anomalies and fetal or infant death (12, 13). Harrison et al. (3) reported two FAAP100 variants with distinct outcomes. Affected infants in family 1, in which the parents were first cousins, were homozygous for a frameshift variant (FAAP100:p.E384Gfs*28) that caused complete protein loss in two live-born males who died in the neonatal period (individual A: died on day seven and individual B: died on day zero). Despite identical mutations, phenotypic variability was still evident. Both infants had multiple congenital anomalies of the heart, brain, kidneys, reproductive system, lower gastrointestinal (GI) tract, and skeletal defects, but individual B also had severe lung and bladder abnormalities. The affected individuals from family 2, a non-consanguineous family, were homozygous for a stop-gain variant resulting in a truncated protein missing 18 amino acids (FAAP100:p. Gln864*). Though they arguably exhibited milder anomalies compared with the affected members of family 1, the mother also experienced two spontaneous first-trimester pregnancy losses in which the fetuses were not tested. Individual C showed heart, kidney, and skeletal abnormalities and died from respiratory failure after febrile seizures at 14 months of age. The parents elected to terminate the pregnancy — individual D — following a 21-week ultrasound that revealed a more severe phenotype than that of individual C, with brain, pancreatic, kidney, and lung abnormalities.

Kuehl et al. (4) studied seven individuals with unclassified FA-like phenotypes that also featured a deficiency in FANCD2 monoubiquitylation. The homozygous FAAP100:p.Thr542Pro missense variant was observed in the offspring of first cousins. The pregnancy was terminated because of severe congenital abnormalities including growth retardation, radial ray defects, duodenal atresia, ventricular septal defect, hydrocephalus, and cellular hypersensitivity to ICL induction. Despite its low predicted pathogenicity and a lack of conservation, functional studies confirmed pathogenicity (4).

## Rigorous functional evidence confirms pathogenicity in rare disease

Harrison et al. (3) and Kuehl et al. (4) and established patient cell lines harboring homozygous *FAAP100* mutations to confirm FA diagnoses. As is typically required for a diagnosis of FA, the authors demonstrated that all *FAAP100* variants caused MMC-induced chromosome breaks, accumulation of metaphase with high breakage rates, and frequent radial figures. Like FANCA and FANCB FA patient cells, the affected cells were deficient in subnuclear FANCD2 foci and FANCD2 monoubiquitination. Cellular phenotypes were rescued by WT FAAP100 complementation via overexpression, providing evidence that the cellular phenotypes were FAAP100 driven. Harrison et al. (3) induced overexpression of the stop-gain mutation from family 2 into patient-derived cells from family 1, in which the frameshift variants lead to loss-of FAAP100, and found that it failed to rescue MMC hypersensitivity, FANCD2 foci formation, or ubiquitination, indicating the stop-gain mutation also resulted in loss of function. These results, while robust, are from a limited number of consanguineous patients and *FAAP100* genotypes, which may introduce confounding genotypic background factors, and cDNA overexpression can also have unintended consequences, making it difficult to attribute observed effects solely to the variants and genes of interest.

Kuehl et al. (4) used multiple FAAP100 functional models, including mouse and zebrafish. FAAP100:p.Thr542Pro disrupts B-strand–mediated binding to FANCB, resulting in a failure of nuclear translocation by FANCA and FANCM (5) and an inability to stimulate FANCL-mediated monoubiquitylation of FANCD2, abolishing the function of the catalytic core complex essential for DNA repair pathway activation. Primary cell cultures from a *faap100^–/–^* zebrafish model were characterized using cell growth studies, chromosomal breakage, cell cycle, and survival analyses, all of which showed defects typical of FA. The *faap100^–/–^* zebrafish exhibited a complete female-to-male sex reversal phenotype consistent with other zebrafish *fanc* knockouts. The authors also generated a *Faap100^–/–^* mouse, which produced smaller *Faap100^–/–^* pups at sub-Mendelian ratios, suggesting embryonic lethality. The *Faap100^–/–^* male mice exhibited testicular atrophy with empty, atrophic seminiferous tubules and absent sperm, whereas the females had ovarian hypoplasia with no follicular differentiation. The findings were indicative of sexual differentiation with impaired germ cell formation, mirroring reproductive defects in other FA models. Genomic instability resulting from inactivation of ICL repair has been demonstrated to lead to apoptosis of primordial germ cells before entry into meiosis, as a quality control mechanism for maintaining the integrity of the germline (14). No blood count abnormalities were observed in the short study period; it will be interesting to perform a detailed analysis of the stem cell compartments and response to stressors such as MMC and 5-fluorouracil. Overall, the *Faap100* mouse model exhibits recurring features observed in other FA mouse models (4, 15).

## Future directions

Cancer predisposition is a key feature of many FAs. The early lethality and small number of patients with *FANCX* or other rare FAs limit the observation of embryonic tumors, such as those that occur with *FANCD1* (aka *BRCA2*) and *FANCN* (aka *PALB2*) (16) or later-onset cancers. Similarly, whether *FAAP100* in heterozygosity predisposes individuals to cancer, as is observed for many members of the FA pathway, particularly downstream members involved in homologous recombination (17, 18), remains unknown. The in utero or neonatal fatality of *FAAP100*-associated cases may explain its delayed identification as an FA gene. It is also possible that there are additional pathogenic variants contributing to the presenting phenotypes. Expanding genomic analyses in perinatal death cohorts will likely identify additional *FANCX* and FA cases, helping define the clinical spectrum.

Current technologies used in genetic diagnostics also enable disease gene discovery when paired with dissemination, data-sharing, publication, and appropriate functional experimental evidence. Clinical Genome Resource (ClinGen) has guidelines to establish robust gene-disease relationships for rare disorders that quantify both genetic and functional evidence (19). Harrison et al. (3) and Kuehl et al. (4) provide conclusive genetic and functional evidence that *FAAP100* is an FA gene ready for diagnostic investigations.

## Author contributions

All authors contributed equally to the preparation of this Commentary and are listed in alphabetical order.

## Version 1 06/02/2025

Electronic publication
